# Time trends of chest pain symptoms and health related quality of life in coronary artery disease

**DOI:** 10.1186/1477-7525-5-13

**Published:** 2007-03-06

**Authors:** Anna Kiessling, Peter Henriksson

**Affiliations:** 1 Karolinska Institutet, Department of Clinical Sciences, Danderyd Hospital, Stockholm, Sweden

## Abstract

**Background:**

There is at present a lack of knowledge of time trends in health related quality of life (HRQL) in common patients with coronary artery disease (CAD) treated in ordinary care. The objective of this study is to assess and compare time trends of health related quality of life (HRQL) and chest pain in patients with coronary artery disease.

**Methods:**

253 consecutive CAD patients in Stockholm County, Sweden – 197 males/56 females; 60 ± 8 years – were followed during two years. Perceived chest pain symptoms and three global assessments of HRQL were assessed at baseline, after one and after two years. EuroQol-5 dimension (EQ-5D) with a predefined focus on function and symptoms; the broader tapping global estimates of HRQL; EuroQol VAS (EQ-VAS) and Cardiac Health Profile (CHP) were used. Chest pain was ranked according to Canadian Cardiovascular Society (CCS). Change in HRQL was analysed by a repeated measurements ANOVA and chest pain symptoms were analysed by Friedman non-parametric ANOVA.

**Results:**

Perceived chest pain decreased during the two years (p < 0.00022); CCS 0: 41–51%; CCS 1: 19–15%; CCS 2: 31–27%; CCS 3: 5–4% and CCS 4: 4–2%. By contrast, HRQL did not change: EQ-5D: 0.76 (CI 0.73–0.79) -0.78 (CI 0.75–0.81), EQ-VAS: 0.68 (CI 0.66–0.71)-0.68 (CI 0.65–0.71) and CHP: 0.66 (CI 0.64–0.69) -0.66 (CI 0.64–0.69).

**Conclusion:**

HRQL did not increase despite a reduction in the severity of chest pain during two years. This implies that the major part of HRQL in these consecutive ordinary patients with CAD is unresponsive to change in chest pain symptoms.

## Background

Disease specific and all-cause mortality in patients with coronary artery disease (CAD) have declined during the last decades [1]. Patients with CAD have at present well treated physical symptoms and cardiovascular drugs help to reduce their risk of a new coronary event. However, the patient's perception of disease is to a large extent unexplored. Health related quality of life (HRQL) is often added as an outcome variable in randomised intervention clinical trials with the aim to assess the patient's perception of the disease. HRQL is – in contrast to morbidity and mortality – a multidimensional construct without a golden standard or definition. HRQL is always assessed and defined by the individual patient. Objective – e g patency – and subjective – e g HRQL – results are often in conflict [2,3]. Some studies report improved perceived function and wellbeing after an intervention resulting in similar levels of HRQL as in normative populations [4,5]. By contrast, in other studies patients examined e. g. four years after an acute myocardial infarction still had a decreased HRQL as compared to community norms [6]. A problem with randomised controlled trials is that they often include highly selected patient groups. This selection decreases the ability to extrapolate the effects, to the unselected cohorts in routine care. Descriptive studies of unselected populations with CAD receiving routine clinical care are scarce. Studies examining time trends in such populations are consequently even more scant. However, in order to understand the patient perspective – illness – of CAD it is mandatory to study HRQL in unselected cohorts of patients. The aim of our study is to assess time trends in chest pain symptoms and HRQL in routine care patients with CAD.

## Methods

### Design and subjects

We identified a cohort of consecutive patients with CAD and assessed chest pain symptoms and perceived quality of life during two years. This was done in connection with a randomised study showing that participatory learning of secondary prevention for general practitioners decreased cholesterol levels in their patients with coronary artery disease [7].

The Department of Medicine at Södertälje Hospital, located in the southernmost part of Stockholm County, Sweden, provides planned and emergency health care for acute and elective cardiac patients in a catchments area of approximately 95 000 habitants. All in- and outpatients with an age less or equal to 70 years who had visited the department during the preceding year, due to symptoms and signs of CAD (ICD-9 code 410–414) were identified (1995). The age limit was chosen due to lack of secondary preventive studies in older patients at time of recruitment. 429 patient records were investigated. 106 patients were excluded, the majority because they did not fulfil the criteria of CAD and a few because of other life threatening disease or because they lived outside the catchments area. 68 of the remaining 323 patients fulfilling the criteria for inclusion refused to participate.

Criteria for a confirmed diagnosis of CAD in the patient record were:

I: A diagnosis of angina pectoris, either by objective criteria based on coronary angiography, or a pathologic exercise- or stress-test, or a clinical assessment based on typical angina symptoms at exercise with or without ECG-evidence of possible or definite ischemia.

II: A diagnosis of myocardial infarction based on either WHO-criteria [8] or on unequivocal ECG-findings.

Hypertension was considered to be present if systolic blood pressure was > 140 mm Hg, if diastolic blood pressure was > 90 mm Hg or if antihypertensive medication was used. Diabetes mellitus was defined as fasting plasma glucose of ≥ 7.0 mmol/l, a self-reported history of diabetes mellitus or treatment for diabetes.

**Figure 1:**
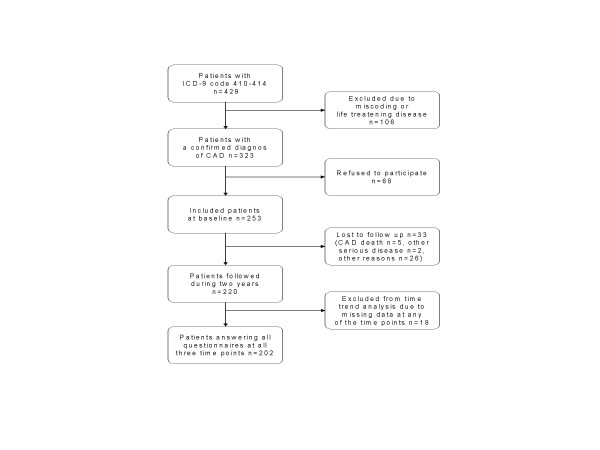
Trial profile. Inclusion, participation, dropouts and follow up.

The patients were examined and they answered questionnaires including symptoms of angina pectoris and HRQL assessments at inclusion, after one respectively after two years. Baseline characteristics are given in Table 1 and the trial profile in figure 1. Further details of the patient cohort, the inclusion procedure and two of the health related quality of life instruments used in this study; the Cardiac Health Profile questionnaire (CHP) and the EuroQol-VAS instrument (EQ-VAS) are available in a previous publication [9]. There were no significant differences in assessed total CHP scores between patients with or without a prior myocardial infarction or between those who had or had not passed an intervention – Percutaneous Coronary Intervention (PCI)/Coronary Artery Bypass Surgery (CABG) – at baseline. Furthermore, a regression analysis showed no influence on HRQL of duration of CAD or age [9].

**Table 1:** Patient characteristics at baseline

Patients included (n)	253
Age (years)	60.1 ± 7.5
BMI (kg/m^2^)	28 ± 4.2
Waist/hip ratio	0.95 ± 0.07
Systolic blood pressure (mmHg)	139 ± 20
Diastolic blood pressure (mmHg)	84 ± 9
Duration of CAD (years)	6.0 ± 5.6
Total lipoprotein cholesterol	6.4 ± 1.1
Triglycerides	2.1 ± 1.1
High density lipoprotein cholesterol	1.2 ± 0.3
Low density lipoprotein cholesterol	4.2 ± 1.0
Men/Women	197 (78%)/56 (22%)
Family history of CAD	97 (38%)
Diabetes mellitus	37 (15%)
Hypertension	67 (26%)
Smoking	
Non smoker	107 (42%)
Ex-smoker	85 (34%)
Current smoker	61 (24%)
History of myocardial infarction	167 (66%)
History of coronary artery bypass grafting	95 (38%)
History of PCI	29 (11%)
History of peripheral arterial disease	5 (2%)
History of cerebrovascular lesions	3 (1%)
Use of Acetyl salicylic acid	205 (81%)
Use of β-blockers	165 (65%)
Use of Lipid lowering drugs	49 (19%)
Other co-morbidity*	71 (28%)

Values are given as mean ± SD or n (%); BMI, Body Mass Index; CAD, Coronary Artery Disease; PCI, Percutaneus Coronary Intervention; *For example locomotive, gastro intestinal, pulmonary, depression, renal calculi and prostate disorders

The HRQL-questionnaires were self-completed. The Swedish versions of the CHP- and EQ-instruments were used. One research nurse performed all interviews and physical examinations at the department. She also handled all case report forms. All participating patients gave written informed consent. The study complies with the Declaration of Helsinki and was approved by the ethics committee of Karolinska Institutet at Huddinge University Hospital.

### Assessment of chest pain symptoms and health related quality of life

The patients ranked existence and degree of current angina symptoms according to the Canadian Cardiovascular Society classification (CCS) 0–4 [10] at baseline, after one respectively two years (Table 2).

**Table 2:** Prevalence and severity of chest pain symptoms ranked according to the Canadian Cardiovascular Society classification (CCS 0–4)

n = 202		Baseline	After 1 year	After 2 years	p < 0.00022*
**CCS 0**	No chest pain	82 (41%)	105 (52%)	104 (51%)	
**CCS 1**	Ordinary physical activity does not cause angina. Strenuous, rapid, or prolonged exertion causes angina	39 (19%)	28 (14%)	30 (15%)	
**CCS 2**	Slight limitation of ordinary activity (special circumstances) due to angina	62 (31%)	54 (27%)	55 (27%)	
**CCS 3**	Marked limitation in ordinary physical activity due to angina	11 (5%)	10 (5%)	9 (4%)	
**CCS 4**	Unable to perform any physical activity without discomfort – angina may be present at rest.	8 (4%)	5 (2%)	4 (2%)	

Friedman non-parametric ANOVA

The definition of HRQL in this study is that 'quality of life' in clinical medicine represents the functional effect of an illness and its consequent therapy upon a patient, as perceived by the patient [11].

The Cardiac health profile questionnaire (CHP) is a disease-specific HRQL questionnaire specially designed for respondents with CAD. CHP assesses the HRQL in a broad perspective. The questionnaire includes questions about physical function/general health, emotional, social and cognitive function. The 16 items in the CHP questionnaire (English version) are shown in Table 3. For further details of content, development, validity, reliability and sensitivity in Swedish cohorts of patients with CAD see previous publications [9,12,13]. The CHP yields an estimate of HRQL between 0 and 100 were 0 is equal to full health and 100 is equal to worst imaginable state. We have in this study transformed the individual global CHP mean scores to a 0–1 scale were 0 is equal to death and 1 is equal to full health. This was done in order to facilitate comparison to the EuroQol results.

**Table 3:** Items in the Cardiac Health Profile questionnaire (CHP)

**Item**	
1	How do you cope with tasks that require concentration and reflection?
2	Are you an active person, full of initiative or passive and listless?
3	Do you easily forget things in the immediate past or where, for example, you have placed things?
4	Do you easily understand and solve problems, make decisions adapt to new situations?
5	Do you feel depressed or have difficulty finding pleasure in things you used to find pleasant?
6	Do you easily become irritated, sad, worried, or anxious?
7	Do you often experience fear, uneasiness or anxiety?
8	Do you easily lose control over your feelings?
9	Are you satisfied with your sleep (quality of sleep, ability to fall asleep, etc.)?
10	Do you have a good relationship to those connected to you (family and friends)?
11	Are you satisfied with your daily life (at work, as a pensioner, as a housewife, as a student, etc.)?
12	Do you experience your leisure time as meaningful and enriching?
13	How is your sexual life?
14	Are you satisfied with your physical capacity to accomplish things you wish to do?
15	How do you experience your general health status?
16	Are you troubled by various kinds of pain other than your known anginal chest pain?

The answers are marked on a 100 mm VAS-scale with verbal anchors expressing extremes. High scores indicates a bad Health Related Quality of Life

Multivariate explorative factor analytical methods were used to identify the underlying structure as described in a previous publication [9]. Four independent principal components – sub-domains – of HRQL were identified. These components are physical function/general health, emotional, social and cognitive function.

The generic HRQL questionnaires EuroQol-5 Dimensions (EQ-5D) self-classifier and EuroQol-VAS (EQ-VAS) were used to test the robustness of the analysis of time trends of HRQL as measured by the CHP questionnaire. In the EQ-5D self classifier the respondents classified their own health status in five aspects – mobility, self-care, usual activities, pain/discomfort and anxiety/depression into one of three levels – no problems, moderate problems and severe problems [14,15]. Each obtained individual health state was then given an index value (0–1) based on a method described by Dolan [16]. Since there is no Swedish tariff for EQ5D-index, the UK EQ5D-index tariff was used to calculate the EQ5D-index values, were 0 is equal to death and 1 is equal to full health [16]. Health states worse than death were given a score of 0 since the scaling of negative health states are controversial [17,18].

In the EQ-VAS method the respondent marks present HRQL on a 20 cm vertical measurement scale with labelled anchors 'dead' (0) and 'full health' (100). The individual numbers marked on the scale were divided by 100 in order to obtain EQ-VAS scores between 0 and 1. Both EQ5D methods are easy and rapid generic instruments, well validated and found to be reliable in different cultures and in different diseases including a new validation study in patients after myocardial infarction [19,20].

### Statistics

The main outcome variable in the present study is change in HRQL and chest pain (CCS) over time. HRQL was analysed by a repeated measurements ANOVA and chest pain symptoms were analysed by Friedman non-parametric ANOVA. The underlying structure of HRQL as assessed by CHP was explored by the factor analytic multivariate technique, principal component (PC) analysis. The 16 item variables were reduced into principal components, i.e. to avoid an influence of multicollinearities and to find orthogonal (independent) factors explaining the variance in the sample [21,22]. Another way to put this is to find independent underlying factors explaining the variation in answers. Varimax rotation was used. The Kaiser criterion was used to demarcate the total number of factors (principal components) to retain. Only factors with Eigen values greater than 1 were retained; i.e. unless a factor extracts at least as much as the equivalent of one original variable, it was dropped. We have described the details in a previous publication [9]. The factor coefficients of the four extracted PC at baseline were used to calculate PC:s at one respectively two years. The coefficients and the formula used can be obtained from the corresponding author. We used Spearman Rank order correlation analysis to assess whether a co variation between chest pain symptoms and HRQL existed. The descriptive data for the HRQL measurements are given as mean and 95% confidence interval. The patient's baseline characteristics are given as mean ± SD or n (%). The power of the present study is 80% to detect a change in EQ5D by 5%.

## Results

The characteristics of the study population at baseline are described in Table 1. 253 consecutive patients (197 males and 56 females) with a mean age of 60.1 ± 7.5 years were included. The response rates were 99% in all of the used HRQL instruments at baseline. The response rate was 98% on CCS. During the study five patients died and all of those due to cardiovascular disease. In addition, two patients had to be excluded due to another serious disease and eight patients had moved out of the district and 18 patients refused participation (figure 1). Thus, we had altogether 220 patients at the two years follow-up (88%). 202 patients answered CHP, EQ 5D, EQ-VAS and CCS at all three assessments points (81% of all included patients at baseline). There were no significant differences in baseline characteristics between responders and non-responders.

### Time trends

**Figure 2:**
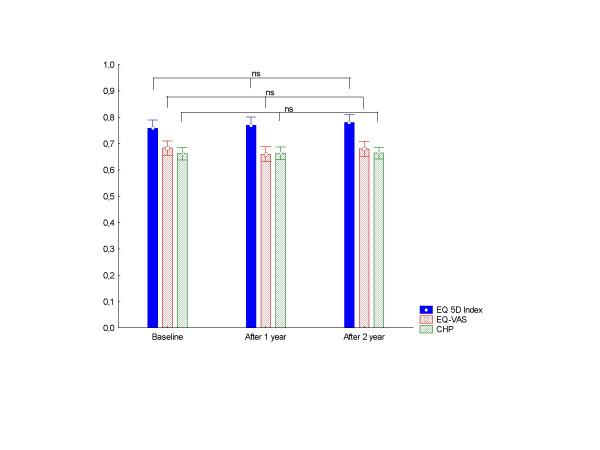
Health related quality of life at different times of follow up assessed by Cardiac Health Profile (CHP), EuroQol-VAS (EQ-VAS) and EuroQol-5 Dimension (EQ 5D-Index) instruments. Time trend during the two years was analysed by a repeated measurement ANOVA.

The prevalence and severity of chest pain symptoms – according to CCS grade – decreased during the two study years as shown in Table 2 (p < 0.00022). By contrast, no significant change occurred in HRQL during the two years, as assess by three different instruments (figure 2). Furthermore no change at all was noted in any of the four major components of HRQL – cognitive, physical/general health perception, social and emotional function – derived from the CHP-instrument; (data not shown). During the study 67 (33%) of the patients contracted non cardiovascular co-morbidity. Analysis with inclusion or exclusion of these 67 patients did not change our results.

An analysis of co-variation between chest pain symptoms and HRQL showed significant correlations between CCS and all three global assessments of HRQL at all three time points, Table 4 (p = 0.000000).

**Table 4:** Correlation between chest pain symptoms and three different global measures of health related quality of life (HRQL) respectively to four independent domains of HRQL

**n = 202**		**Spearman Rank**	**t(N-2)**	**p-value**
**EQ-5D_Index_**	**Baseline**	-0.58	-10.02	0.000000
	**After 1 year**	-0.60	-10.53	0.000000
	**After 2 years**	-0.64	-11.82	0.000000
**EQ_VAS_**	**Baseline**	-0.48	-7.66	0.000000
	**After 1 year**	-0.52	-8.64	0.000000
	**After 2 years**	-0.48	-7.78	0.000000
**CHP**	**Baseline**	-0.39	-6.06	0.000000
	**After 1 year**	-0.45	-7.22	0.000000
	**After 2 years**	-0.35	-5.33	0.000000
**PC_cognitive_**	**Baseline**	0.05	0.70	0.48
	**After 1 year**	0.10	1.39	0.17
	**After 2 years**	0.09	1.30	0.20
**PC_physical/general_**	**Baseline**	0.41	6.42	0.000000
	**After 1 year**	0.47	7.45	0.000000
	**After 2 years**	0.43	6.66	0.000000
**PC_social_**	**Baseline**	0.11	1.63	0.10
	**After 1 year**	0.23	3.32	0.001
	**After 2 years**	0.08	1.14	0.25
**PC_emotional_**	**Baseline**	0.18	2.65	0.009
	**After 1 year**	0.21	3.09	0.002
	**After 2 years**	0.15	2.08	0.04

Spearman Rank Order Correlation between existence and severity of current chest pain symptoms assessed by Canadian Cardiovascular Society classification CCS (0–4) and different measures of health related quality of life

EQ-5D_Index _denotes EuroQol 5 Dimension questionnaire transformed to an Index score (0–1)

EQ_VAS _denotes EuroQol-VAS instrument (0–1)

CHP denotes the Cardiac Health Profile questionnaire (global mean score)

PC denotes the four independent domains of HRQL calculated from the CHP questionnaire

**Figure 3:**
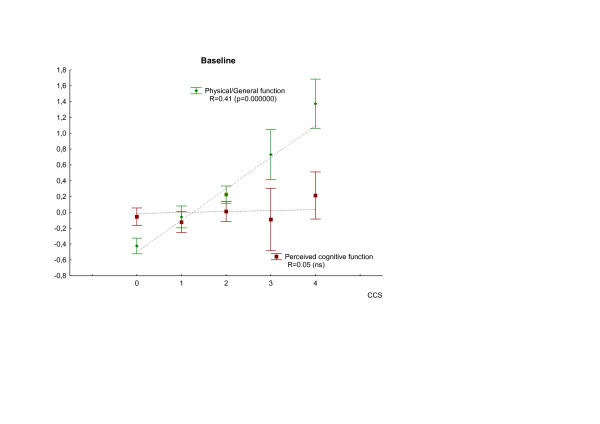
Covariation of chest pain grade and and the physical/general health and cognitive domains of health related quality of life at baseline. Chest pain is assessed by Canadian Cardiovascular Society classification (CCS; 0–4). Values are given as mean ± SE. Spearman Rank order correlation analysis was used.

**Figure 4:**
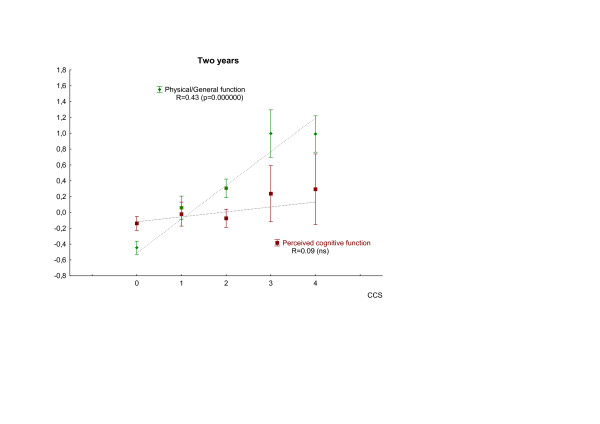
Covariation of chest pain grade and the physical/general health and cognitive domains of health related quality of life after two years. Chest pain is assessed by Canadian Cardiovascular Society classification (CCS; 0–4). Values are given as mean ± SE. Spearman Rank order correlation analysis was used.

A similar analysis of the four independent sub-domains – principal components – showed a correlation between chest pain symptoms and the physical/general health domain at all three time points (p = 0.000000). By contrast, there was no correlation between cognitive function respectively social function domains and chest pain symptoms at any of the time points (Table 4). Figure 3 and 4 illustrates the different patterns of co-variation between chest pain and the cognitive respectively physical/general health domains of HRQL at baseline and at two years.

## Discussion

The prevalence and severity of chest pain symptoms decreased during two years of follow-up as assessed by CCS grade in this unselected cohort of patients with CAD. However, no change was found in global HRQL as assessed by three different HRQL instruments despite the symptom reduction. This indicates that an important part of HRQL in such patients is unresponsive to change in the existence or degree of chest pain. Several explanations to this could be discussed. One possible confounder could be a change in non-cardiac morbidity. However, a separate analysis excluding patients contracting co-morbidity during the study did not change the results. The reason for the unresponsiveness could be the multidimensional construct of HRQL. As shown in table 4 the CCS grade seems to explain slightly more than one third of the variation in EQ-5D and one sixth of the variation in CHP at each time point. Looking at the main components of HRQL, CCS explained slightly less than one fifth of the variation in physical function, a minor fraction of the variation in emotional function and none of the variation in cognitive and social function at all time points. However, none of these components changed during the time period.

We have in a previous paper found that perceived cognitive function explains the major part of HRQL in this cohort [9]. However, the result of the correlation analysis showed no relation between perceived cognitive function and chest pain symptoms. Studies exploring cognitive function in CAD – either objectively measured or as perceived by the patient – are scarce. We conclude that important parts of HRQL in these patients do not depend on chest pain symptoms. This finding is strengthened by another study [23]. The effect of coronary artery bypass graft surgery was assessed by the Medical Outcome Study Short Form-36 (SF-36) questionnaire. HRQL improved in 6 of 8 subscales. However, general health and role emotional scores did not improve in parallel to the decrease in chest pain symptoms. This finding indicates that major parts of HRQL – in patients with CAD – do not improve in parallel to an improved physical function.

A limitation of our study is that that we just assessed perceived function. However, the main aim was to evaluate time trends in illness – both in terms of HRQL and chest pain symptoms – and not primarily to address a comparison between objective function and subjective perception. Furthermore it is known that the correlation is weak between objectively measured cognitive function and perceived cognitive function [24].

Strength of our study is that HRQL was assessed both by three different global instruments and by the independent sub-domains. The response rate was also high. This should increase the robustness of our analysis and conclusions. Another strength is that this large sample of patients were followed over a good [2-year] period. Furthermore, the cohort was unselected and included both patients with and without present physical symptoms. This should increase the ability to understand the comprehensiveness of different assessment instruments and to analyse a putative co variation of change in HRQL and other estimates over time.

The comprehensiveness differs between the three HRQL instruments. EQ-VAS is an individually defined global estimate. The 16 items of the CHP questionnaire taps – as compared to other frequently used instruments – a broad perspective of aspects on HRQL. It includes e g questions concerning the ability to select relevant information, and to understand, retain, express and apply knowledge in specific contexts of life. In a previous analysis we showed that questions representing cognitive function explained 43% of the HRQL variation [9]. EQ-5D includes five questions tapping five defined aspects of HRQL. It has been demonstrated that adding a cognition attribute to the standard EQ-5D system increases its comprehensiveness [25].

There was a slight but insignificant improvement of HRQL as assessed by EQ-5D (2.6%; p = 0.28). However, the power analysis showed that we could exclude a more clinically relevant improvement in HRQL by 5% or more even in the symptom driven EQ-5D. EQ-5D also showed a better global HRQL by 0.1 (p = 0.0000) than CHP and EQ-VAS. An explanation might be the more narrow scope of EQ-5D tapping mainly physical and emotional aspects of HRQL.

It is known that physicians tend to underestimate or fail to recognise functional disabilities that are reported by their patients, especially disabilities related to social activities [26]. The physician has a disease perspective and translates the absence or decrease of chest pain symptoms to less stenotic coronary arteries with improved capacity to distribute oxygen to fulfil the demand of the working myocardial tissue. This should improve the patients' physical function. However, the patients' perception of health might have a great inertia to respond to decreased chest pain symptoms. All patients in the present study knew that their coronary arteries were affected by atherosclerosis. To many patients this knowledge might mean that they constantly are afflicted by a potentially lethal condition.

A limitation and strength of the present study is of course the unselected sample in concert with the fact that the patients are included from a single geographic area. The study addresses an important issue about responsiveness of HRQL instruments. Our findings of a low sensitivity of the HRQL instruments to change in symptom burden is interesting and challenging both from a clinical and societal perspective. The results should stimulate future research on the complex relations between objectively measured disease and subjectively perceived illness for patients with CAD.

## Conclusion

The major part of assessed health related quality of life is unresponsive to a decrease in severity of chest pain symptoms in a consecutive cohort of ordinary patients with coronary artery disease. This indicates that a reduction of chest pain symptoms is not sufficient; to improve health related quality of life in patients with coronary artery disease. The dominating dimension of health related quality of life – cognitive function – does not differ between patients with or without chest pain symptoms. The low responsiveness of assessed health related quality of life outcome to a change in symptom burden is interesting and challenging both from a clinical and a public health perspective.

## Competing interests

The author(s) declare that they have no competing interests.

## Authors' contributions

PH and AK shared the conception and analysis of the study. AK was responsible for the statistical analysis, drafting and revising of the article. PH and AK shared the hypothesis and design. PH and AK are both guarantators.
